# AMP Challenge: towards a standardized, community-wide benchmark for AI-driven antimicrobial peptide design

**DOI:** 10.1186/s13059-026-04316-3

**Published:** 2026-11-14

**Authors:** Ewa Szczurek, Paulina Szymczak, Rasmus Møller-Larsen, Bruno Puczko-Szymański, Marcelo D. T. Torres, Cesar de la Fuente-Nunez, the AMP Challenge Organizing Committee

**Affiliations:** 1 Institute of AI for Health, Helmholtz Munich, Neuherberg, Germany; 2 Faculty of Mathematics, Informatics, and Mechanics, University of Warsaw, Warsaw, Poland; 3 Faculty of Mathematics, Informatics, and Statistics, Ludwig-Maximilians-Universität München, Munich, Germany; 4 Munich Center for Machine Learning, Munich, Germany; 5 Faculty of Medicine, Ludwig-Maximilians-Universität München, Munich, Germany; 6 School of Computation, Information and Technology, Technical University of Munich, Garching Bei Munchen, Germany; 7 Machine Biology Group, Departments of Psychiatry and Microbiology, Institute for Biomedical Informatics, Institute for Translational Medicine and Therapeutics, Perelman School of Medicine, University of Pennsylvania, Philadelphia, PA USA; 8 Departments of Bioengineering and Chemical and Biomolecular Engineering, School of Engineering and Applied Science, University of Pennsylvania, Philadelphia, PA USA; 9 Department of Chemistry, School of Arts and Sciences, University of Pennsylvania, Philadelphia, PA USA; 10 Penn Institute for Computational Science, University of Pennsylvania, Philadelphia, PA USA

*Here we introduce the AMP Challenge, a NeurIPS 2026 competition that provides the first standardized, blinded, head-to-head benchmark for generative AI models designing antimicrobial peptides, addressing the field’s urgent need for a shared way to identify which computational strategies actually translate into real antimicrobial activity.*

Antimicrobial resistance is projected to become one of the leading causes of death worldwide, having already contributed to an estimated 4.95 million deaths in 2019 [1]. Antimicrobial peptides (AMPs) are considered one of the most promising alternatives to conventional antibiotics, since bacteria develop resistance to AMPs far more slowly than to conventional drugs. Over the past five years, generative artificial intelligence has transformed how such peptides are designed. Dozens of methods, spanning variational autoencoders, generative adversarial networks, diffusion models, geometric deep learning architectures [2] and large protein language models [3], have now been published across research groups worldwide (see Table 3 in [4] for a broader overview). This growing computational toolkit complements large-scale efforts to mine existing genomes and microbiomes for naturally encoded AMPs [5], and peptides identified either way have already shown promising activity in animal models. Yet despite this proliferation, the field still lacks a shared way to establish which of these methods, if any, reliably produce peptides that work outside their original publication. Resolving this is urgent, because with every additional unvalidated method it is harder to know where to invest computational effort, both for research and ultimately for clinical translation.

In silico metrics, such as novelty, diversity, distributional fidelity or discriminator-predicted activity, are useful, but each captures only one facet of design quality and each has known blind spots. seqme, an open-source library that consolidates sequence-, embedding- and property-based metrics for exactly this purpose, found in a recent benchmark that no single generative model dominates across all criteria [6]. While in silico scores provide important proxies for model evaluation and experimental pre-ranking, only ground-truth wet-lab measurement of minimum inhibitory concentration (MIC) and hemolytic activity (HC_50_) can establish whether a designed peptide actually works. Indeed, accumulating evidence suggests that AI models trained on increasingly large, standardized and high-quality experimentally validated datasets show improved predictive performance and closer concordance between computational predictions and experimental outcomes [7]. In other words, expanding the pool of carefully curated experimental labels may be a key driver of more accurate and reliable AI-guided biological discovery.

However, a compounding challenge is that experimental validation remains inconsistent across the field. Different studies use different bacterial strains, media, inoculum sizes and activity thresholds, while negative training datasets, used to teach discriminative models what an inactive peptide looks like, are often assembled through inconsistent and demonstrably biased procedures [8]. As a result, when one generative method is reported as having a better success rate than another, it is frequently unclear whether that reflects a truly better model, a more permissive assay, or simply more careful, human-guided candidate selection. The literature is accumulating dozens of generative approaches [4], but provides almost no head-to-head comparative evidence, nor does it clarify which computational metrics actually predict real-world antimicrobial efficacy.

The AMP Challenge, part of the NeurIPS 2026 Competition track, organized by the Szczurek group (Helmholtz Munich, University of Warsaw and LMU Munich) and the de la Fuente laboratory (University of Pennsylvania), is designed to close exactly this gap. By giving every participating team the same computational evaluation pipeline, the same experimental protocol, and the same panel of clinically relevant bacterial strains, it makes it possible, for the first time, to compare models, and only models, rather than the many confounding factors that currently separate one published claim of success from another.

The format welcomes any generative approach, including language models, diffusion models, VAEs, GFlowNets, reinforcement learning, Bayesian optimization, evolutionary algorithms, and hybrids of these. Each team is expected to generate a library of 50,000 peptide sequences and to submit a ranked shortlist of their top 100 candidates, together with an abstract describing the method and a summary of training data and any manual filtering applied. Submissions first undergo an automated compliance check for sequence validity, length, and licensing. The full 50,000-sequence libraries will then be screened computationally, using the seqme framework, for diversity, novelty and physicochemical properties, and will be checked against established AMP databases (DBAASP, dbAMP, APD) to quantify novelty. If the number of qualifying teams exceeds experimental capacity, this computational screening is used to rank teams and select the top 20 for wet-lab testing. The final conclusion of the AMP Challenge will take place at the Keystone Progress in Antibiotic Discovery and Development Symposium, 2027.

For experimental validation, 25 peptides will be drawn at random from each advancing team’s top-100 list, yielding a cohort of 500 peptides tested under identical conditions. All peptides will be synthesized through the same vendor workflow and assayed by broth microdilution against a panel of 20 clinically relevant bacterial strains, spanning Gram-negative and Gram-positive species as well as multidrug-resistant (MDR) isolates from the ESKAPEE group, an acronym for *Enterococcus faecium*, *Staphylococcus aureus*, *Klebsiella pneumoniae*, *Acinetobacter baumannii*, *Pseudomonas aeruginosa*, *Enterobacter* species and *Escherichia coli*, seven pathogens widely designated as priority targets for new antibiotics, alongside hemolysis assays. Samples will be blinded to team identity and tested in randomized order, with the same positive and negative controls in every batch. Teams will be ranked in five categories, covering broad-spectrum, Gram-positive, Gram-negative, MDR ESKAPEE and selectivity (safety window) performance, using pre-registered, unambiguous scoring rules.

All advancing teams will receive their full experimental results. Teams that additionally release a public repository with model weights, inference code, a permissive license, a fixed random seed, and full training-data disclosure will qualify for co-authorship on the joint benchmarking paper. The deliverables from this effort are intended for the whole field, not just the participants, and include an open, CC-BY-licensed dataset pairing computational descriptors with standardized MIC and HC_50_ measurements, including unsuccessful as well as successful peptides, to counteract the publication bias that currently plagues the literature; a public repository of participating teams’ models and code; and, we hope, the first empirical answer to the question of which computational metrics, filtering rules and ranking strategies most accurately predict experimental antimicrobial activity.

Participation in the challenge is free, with all peptide synthesis and experimental assays arranged by the challenge organizers, and participants do not need laboratory access or undertake any experiments. Full details of the submission process, the peptide design constraints, the reproducibility requirements, and the current submission timeline are available on the AMP Challenge website (https://szczurek-lab.github.io/amp-challenge-website/) and in the accompanying GitHub template (https://github.com/szczurek-lab/amp-challenge-2027).

Antimicrobial resistance will not wait for our field to agree on best practices. By replacing dozens of incompatible, self-reported claims with one shared, blinded, standardized comparison, the AMP Challenge aims to turn a fragmented body of promising but incommensurable results into cumulative, trustworthy evidence, making it substantially easier for the community to identify which computational approaches are worth pursuing, and to move the most promising AMP candidates from a laptop screen towards the clinic.

## Data Availability

This manuscript does not report new data or materials. Information about the AMP Challenge, including its submission process, peptide design constraints, and reproducibility requirements, is available on the AMP Challenge website (https://szczurek-lab.github.io/amp-challenge-website/) and in the accompanying GitHub repository (https://github.com/szczurek-lab/amp-challenge-2027).
